# Supplementary material for: Reprimo (RPRM) as a Potential Preventive and Therapeutic Target for Radiation-Induced Brain Injury via Multiple Mechanisms
Source: Int J Mol Sci. 2023 Dec 2;24(23):17055. doi: 10.3390/ijms242317055 (PMC10707327; doi:10.3390/ijms242317055)
Supplement: Supplementary file 1 [file ijms-24-17055-s001.zip › ijms-2659290-supplementary.pdf]

Reprimo (RPRM) as a Potential Preventive and Therapeutic Target for Radiation-Induced Brain Injury via Multiple Mechanisms

Zhujing Ye†, Jin Wang†, Wenyu Shi, Zhou Zhou, Yarui Zhang, Jingdong Wang and Hongying Yang*

State Key Laboratory of Radiation Medicine and Protection, School of Radiation Medicine and Protection, Collaborative Innovation Center of Radiological Medicine of Jiangsu Higher Education Institutions, Suzhou Medical College of Soochow University, Suzhou 215123, China;

18306205857@163.com (Z.Y.); 18862187194@163.com (J.W.); shiwenyv@163.com (W.S.); 18260187268@163.com (Z.Z.); yinxiaolei_69@163.com (Y.Z.); wangjindong@suda.edu.cn (J.W.)

* Correspondence: yanghongying@suda.edu.cn; Tel.: +86-512-65882637; Fax: +86-512-65888340

† These authors contributed equally to this work.

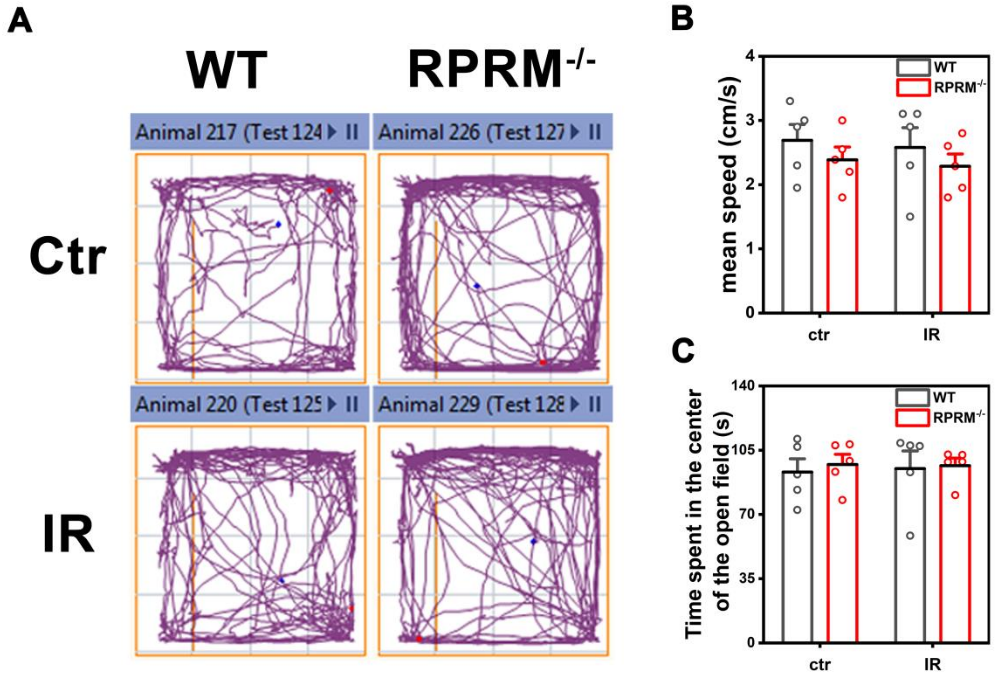

Fig. S1 The open field test on 6–8-week-old WT and RPRM KO male mice after exposed to 10 Gy WBI. **A** Typical exploration tracks of the four groups of mice in the open field test. **B** Mean speed of the four groups of mice in the open field test. **C** Time the four groups of mice spent in the center of the open field. n=5. Data were presented as mean ± SEM. Data were analyzed using two-way ANOVA with Tukey's correction.

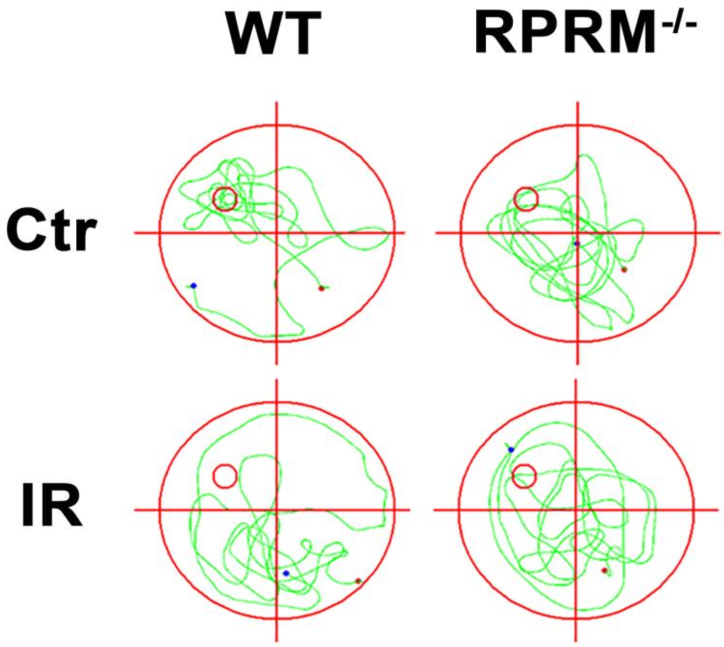

Fig. S2 Representative exploration tracks of 6–8-week-old WT and RPRM KO mice in the spatial probe test of MWM 2 months after exposed to 10 Gy WBI.

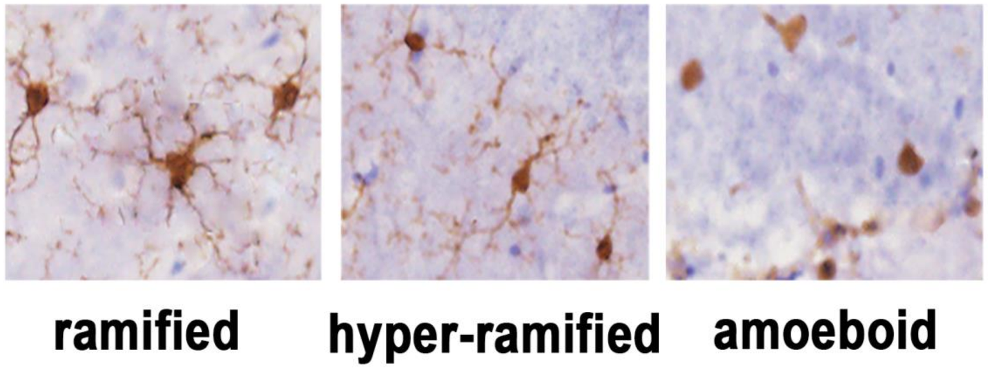

Fig. S3 Representative morphology of microglia in different status including ramified (non-activated), hyper-ramified (reactive) and unramified (activated) in mouse hippocampus.
